# Nanoparticle-Based Therapeutics to Overcome Obstacles in the Tumor Microenvironment of Hepatocellular Carcinoma

**DOI:** 10.3390/nano12162832

**Published:** 2022-08-17

**Authors:** Yuanfei Lu, Na Feng, Yongzhong Du, Risheng Yu

**Affiliations:** 1 Department of Radiology, Second Affiliated Hospital, School of Medicine, Zhejiang University, 88 Jiefang Road, Hangzhou 310009, China; 2 Institute of Pharmaceutics, College of Pharmaceutical Sciences, Zhejiang University, 866 Yuhangtang Road, Hangzhou 310058, China

**Keywords:** hepatocellular carcinoma, tumor microenvironment, immunosuppression, nanoparticles

## Abstract

Hepatocellular carcinoma (HCC) is still a main health concern around the world, with a rising incidence and high mortality rate. The tumor-promoting components of the tumor microenvironment (TME) play a vital role in the development and metastasis of HCC. TME-targeted therapies have recently drawn increasing interest in the treatment of HCC. However, the short medication retention time in TME limits the efficiency of TME modulating strategies. The nanoparticles can be elaborately designed as needed to specifically target the tumor-promoting components in TME. In this regard, the use of nanomedicine to modulate TME components by delivering drugs with protection and prolonged circulation time in a spatiotemporal manner has shown promising potential. In this review, we briefly introduce the obstacles of TME and highlight the updated information on nanoparticles that modulate these obstacles. Furthermore, the present challenges and future prospects of TME modulating nanomedicines will be briefly discussed.

## 1. Introduction

Hepatocellular carcinoma (HCC) remains the most commonly diagnosed type of primary liver cancer [1]. According to the global cancer statistics 2020, HCC ranks sixth in terms of incidence and third in terms of mortality rates [1]. HCC accounts for ~80–90% of patients diagnosed with cirrhosis [2]. Therefore, the high prevalence of liver dysfunction in HCC patients limits the application of different treatments [2,3]. For HCC patients in early-stage and with well-preserved liver function, surgical approaches, including resection, ablation, and liver transplantation, are the possible curative options. However, high recurrence rates after resection locally continue to be a major obstacle [4]. Only a small subgroup (~15%) of patients are eligible for surgery, with a 5-year survival rate of 33–50% [5]. For the majority of patients found in the advanced stage, loco-regional and systemic therapies are the treatments of choice [6]. Sorafenib and Lenvatinib are the first Food and Drug Administration (FDA)-approved first-line therapies for advanced and unresectable HCC [7]. Unfortunately, because of overexpression of the multidrug resistance genes, HCC is inherently a chemotherapy-resistant tumor [8,9].

The management of cancer has changed dramatically since the rapid development of systemic treatments with immune therapies [10]. Immunotherapy, which employs immune cells to boost natural defenses to assault cancer cells, has achieved significant advances over the decades [11]. Nivolumab, the anti-PD-1 monoclonal antibody, has been approved by FDA for HCC immunotherapy [12]. In the CheckMate 040 study, nivolumab treatment showed durable responses and hopeful long-term survival in sorafenib-experienced patients with advanced HCC [13]. Though several major types of immunotherapies, including immune checkpoint inhibitors, cancer vaccines, adoptive cell transfer, etc., show durable anti-tumor effects, the limited response rate is one of the major obstacles in application [14,15], which may attribute to “immunological ignorance” and immune escape in the tumor microenvironment (TME) [16]. The overall objective response rate of nivolumab was approximately 15–20% [17]. Therefore, for better stimulating anti-tumor immunity, it is vital to comprehend and modify the microenvironment of HCC.

Until recently, pharmacological efforts to find new medications have primarily focused on oncogenic signaling networks, but the TME, where cancers originate, has just recently emerged as a prominent target for anti-cancer therapies. When cancer cells invade and alter homeostasis, the TME is formed. The cells of the immune system (e.g., T lymphocytes, dendritic cells (DCs), macrophages and neutrophils, and non-immune components (e.g., extracellular matrix (ECM), fibroblasts, and endothelial cells of vessels) form the TME, which immediately surrounds cancer cells. The TME not only provides a protective “ecological niche” for tumor cells to thrive, progress, and metastasize but also affects the responses to therapy [18,19]. Previous studies have demonstrated that immunosuppressive TME facilitates cancer evasion from immunosurveillance [20,21,22]. With the improved understanding of TME, modulation of TME from an immunosuppressive one toward an immune-promoting one provides a new direction in cancer immunotherapy. Reprogramming or re-educating tumor-promoting and suppressive TME may increase anti-tumor immunity by recognizing antigens by the reawakened immune system.

The TME plays an important role in the efficiency of HCC immunotherapy, which attracts increasing attention and drives TME-based research. Due to the impact of renal clearance and biological barriers, the majority of drugs cannot successfully reach the tumor site [23]. Therefore, nanoparticles are utilized as potential vehicles for medicine delivery for their function of prolonging retention time and targeting agents [24]. On the one hand, the enhanced permeability and retention (EPR) effect facilitates tumor accumulation of nanomedicines [25]. Besides passive medication delivery via nanoparticles, nanoparticles can also be modified to further increase their compatibility and efficacy. For instance, mannose-modified nanoparticles can actively target the mannose receptors on tumor-associated macrophages (TAM) which “re-educated” the TME, thus improving therapeutic efficacy [26]. Various innovative nanoparticle-based drugs targeting components of TME of HCC have emerged, with significant advances in both lab and clinic experiments [27,28,29,30]. This review mainly focuses on applications of different nanoparticles to modulate and reprogram components in TME that are major obstacles to HCC therapy. We first introduce the tumor-promoting components of TME and then discuss the recent achievements of TME modulating nanomedicines, which offer a critical perspective on the future development of TME modulating nanomedicines in HCC.

## 2. Major Constituents of the Tumor Microenvironment

The TME of HCC is a dynamic system, which consists of various types of cells (including cancer cells, immune cells, stromal cells, etc.), ECM, vasculature, and other secreted molecules [31,32]. Below, we describe the major components that are major obstacles to HCC therapy.

### 2.1. Abnormal Vasculature of TME

Like other solid tumors, the growth and progression of HCC will induce tumor angiogenesis in order to supply oxygen and nutrients during this period. Unlike normal vessels, tumor vessels are aberrant in structure and function, which impair blood perfusion in tumors, spatially and temporally [33]. The resulting hypoxia not only promotes tumor progression and metastasis by changing the gene expression of tumor cells but confers resistance to therapy [34,35], thereby creating a vicious cycle. The enhanced interstitial fluid pressure and inadequate perfusion caused by these leaky blood vessels increase the number of immunosuppressive cell types and decrease the delivery of therapeutic medications to the tumor [36]. HCC treatment can involve angiogenesis as a target. Major pro-angiogenic factors include but are not limited to vascular endothelial growth factor (VEGF)-A, basic fibroblast growth factor (bFGF), and interleukin-8 (IL-8) [37]. Accumulating evidence indicates that increased VEGF levels in HCC are related to tumor angiogenesis and progression [38].

### 2.2. Cancer-Associated Fibroblasts and ECM

Cancer-associated fibroblasts (CAFs) constitute a dominant cellular component of the TME, which act as key players in the development of tumors and cancer cell evasion of therapies [39]. CAFs release a variety of ECM proteins (such as type I-V collagen and fibronectin), paracrine factors, cytokines, and vasculogenic mimicry, all of which aid in the start of HCC with a malignant character [40]. CAFs secrete angiogenic factors, including VEGF, bFGF, angiopoietin-1(ANG-1), and ANG-2, which induce neovascularization [41]. ECM is mainly produced by CAFs, which act as a scaffold in the tumor [42]. ECM undergoes extensive remodeling during cancer progression with characteristics of stiffness and degradation [43]. ECM stiffness is a physical barrier to the efficient absorption or transport of drugs to deeper regions of the tumor [44].

### 2.3. Immunosuppressive Immune Cells in TME

Tumor-associated immune cells can both assist and impede therapeutic efficacy, and their activation status and location within the TME can vary. Representative immunosuppressive immune cells are the focus of this essay.

#### 2.3.1. Myeloid-Derived Suppressor Cells

Myeloid-derived suppressor cells (MDSCs) are immature myeloid cells with heterogeneity and immunosuppressive properties that are important components of the suppressive TME. MDSCs can be divided into two subsets: monocytic (M-MDSC) and polymorphonuclear (PMN-MDSC). M-MDSCs are more prevalent in tumors and have greater suppressive activity than PMN-MDSCs [45]. MDSCs induce immunosuppressive cells, regulatory T cells (Tregs), and M2-polarized TAM (M2-TAMs) or inhibited immune effector cells (CD8^+^ T cells, DCs, NK cells, etc.) by a variety of methods [46]. Infection with the hepatitis B virus (HBV) is the most common risk factor for HCC, accounting for around 50–80% of all cases [47]. Importantly, MDSCs play a crucial role in maintaining immunotolerance to high levels of HBV replication [48]. Considerable evidence that has implicated the abundance of MDSCs could be employed as an independent prognostic and predictor in human HCC [49]. Infiltrated MDSCs in HCC overexpressed two enzymes: ARG1 and iNOS, which deplete the essential amino acid L-arginin for T cells [50,51]. Therefore, MDSCs could be a promising target for reversing the immunotolerant state in HCC.

#### 2.3.2. Regulatory T Cells

A subgroup of CD4^+^ T cells called Tregs is crucial for preserving immunological immune homeostasis and preventing excessive autoimmunity deleterious to the host [52]. In healthy conditions there is an equilibrium between Tregs and T helper 17 cells to keep peripheral tolerance [53]. However, this balance is disturbed in TME. The number of Tregs increases in TME of HCC patients, which links to compromised immune responses [54]. To mediate their suppressive functions, Tregs secrete inhibitory cytokines (such as transforming growth factor-β (TGF-β), IL-10, etc.), promote cytolysis, and “metabolic disruption” of the effector T cells, and inhibit the maturation of DCs [55].

#### 2.3.3. M2-Polarized Macrophages

TAMs are another important component of immune cells in TME, which are broadly classified into M1-TAMs (tumor-suppressing subtype) and M2-TAMs (tumor-promoting subtype) [56]. As opposed to M1-TAMs, M2-TAMs, alternatively activated by TH2 cytokines IL-4/IL-13 [57], facilitate HCC progression by producing mediators that support tumor cell proliferation and immune escape [58,59]. According to several studies, HCC-derived exosomes can activate macrophages and exhibit the M2 phenotype, thereby promoting HCC development [60,61,62]. In theory, reprograming TAMs from M2 to M1 phenotype or eliminating present TAMs may be a considerable therapeutic approach to arouse their anti-tumor efficacy.

### 2.4. Crosstalk in the Dynamic TME

**Figure 1:**
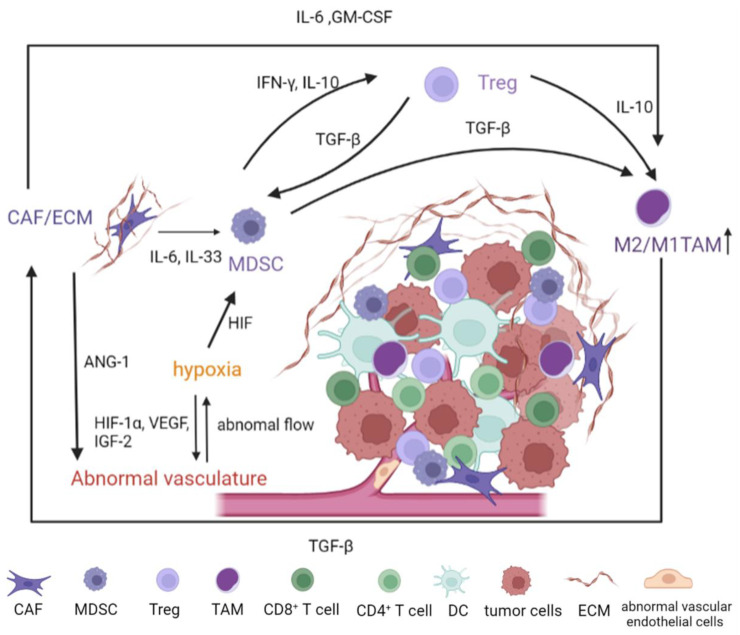
Schematic of the obstacles and their crosstalk in the dynamic TME of HCC. Complex connections affect the growth of HCC and hinder the immune system’s ability to fight it by promoting the activation of immune cells with immunosuppressive qualities. The growth and progression of HCC induce tumor abnormal vasculature and hypoxia, which negatively impacts the infiltration of immune cells and impairs host immunity. Immune suppressive cell types in the TME (MDSC, regulatory Tregs, and M2-TAM) secrete factors that establish immune tolerance to block cancer cell destruction. HCC: hepatocellular carcinoma; TME: tumor microenvironment; CAFs: cancer-associated fibroblasts; ECM: extracellular matrix; TAM: tumor-associated macrophages; MDSC: myeloid-derived suppressor cells; Tregs: regulatory T cells; GM-CSF: granulocyte-macrophage colony-stimulating factor; IL-6: interleukin-6; TGF-β: transforming growth factor-β; IFN-γ: Interferon-γ; HIF: hypoxia-inducible factor; VEGF: vascular endothelial growth factor; IFG-2: insulin-like growth factor-2; ANG-1: angiopoietin-1.

The TME of HCC is a dynamic network and complex connections affect the growth of HCC and hinder the immune system’s ability to fight it by promoting the activation of immune cells with immunosuppressive qualities (Figure 1). For example, the hypoxia induced by abnormal vasculature drives tumor and stromal secretion of pro-angiogenic factors (hypoxia-inducible factor (HIF), VEGF, insulin-like growth factor-2 (IFG-2), etc.) [63]. Most immune cell types have their functions directly or indirectly modulated by hypoxia, which promotes the growth of tumors. IL-10 and interferon-γ (IFN-γ) produced by MDSDs affect Treg induction, while Tregs can, in turn, control the proliferation and function of MDSCs [64]. The CAFs can induce M2-TAMs via secretion of IL-6 and granulocyte-macrophage colony-stimulating factor (GM-CSF) [65]. Therefore, improvements in our knowledge of the local microenvironment of a growing tumor may present greater options for precise drug delivery.

## 3. Nanomedicine-Based Strategies for TME Modulation

Nanotechnology offers a novel opportunity to deliver medicine to the site of the tumor via passive or active targeting ways. In passive targeting, the therapeutic substance is incorporated into a nanoparticle that passively travels to the target organ without a ligand. To increase the preferential accumulation of the drug at the targeted site, active targeting through the conjugation of receptor-specific ligands is a promising approach [66]. Compared with normal cells, some molecules and proteins are upregulated on the surface of HCC cells, such as asialoglycoprotein receptor, gycyrrhizin/glycyrrhetinic acid receptor [67], transferrin receptor [68], folate receptors [69], CD44 [70], and so on. Thus, their ligands can be used to decorate nanoparticles for active targeting. For example, folic acid (FA) can bind to folate receptors on cancer cells with high specificity. In vitro and in vivo data showed that the functional nanodroplets with FA enhance selective accumulation when targeting Hepa1–6 cells more than non-targeting nanodroplets [71]. Another study designed a type of FA-modified Fe_3_O_4_ nanoparticles to specifically co-deliver anti-tumor drugs to HCC [72].

A variety of nanomaterials, including polymeric nanoparticles, liposomes, and metal nanoparticles [73], have gained a lot of attention in potentiating cancer therapies, especially in cancer immunotherapy [74,75,76]. It is noteworthy that parameters such as shape, surface functionalization, and surface charge would have remarkable effects on drug delivery kinetics and biodistribution [77]. Pegylated liposomal formulation Doxil^®^ showed promising activity and low cardiotoxicity compared with doxorubicin (DOX) in metastatic breast cancer [74]. Compared with free DOX, DOX-loaded liposomes significantly increased the uptake of DOX by HCC cells. DOX-loaded liposomes robustly enhanced mild ablation therapy in HCC and represented a viable nanoparticle-based therapeutic approach for HCC treatment [78]. Cubosomes, a type of lyotropic liquid crystalline lipid nanoparticles, are an emerging class of lipid-based nanoparticles. Recently, Pramanik, A. et al. developed Affimer-tagged cubosomes loaded with the anti-cancer drug copper acetylacetonate as a colorectal cancer therapeutic [75], which showed a higher survival rate than the control groups. A recent study revealed that compared with negatively charged PEG-stabilized polymeric nanoparticles, positive ones were better suited for HCC [76]. Furthermore, another study revealed the ability of metal-based ZnS@BSA nanoclusters to facilitate anti-tumor immunotherapy for HCC [79].

Cancer immunotherapy has undergone a revolution over the past decades. The application of nanomedicine has made significant progress in overcoming the constraints of immunological tolerance created by clinic-approved immunotherapies. With the advancement of nanotechnology, an increasing number of intelligent nanomaterials have been designed to re-mode the TME to improve the efficacy of anti-tumor therapies [80]. Since the first nano-drug was approved by the FDA in 1995 (Doxil^®^) [81], more researchers have an increasing interest in exploring novel nanomedicines targeting non-tumoral cells of TME [82], which held great promise in treating primary and metastatic tumors. Hence, in this section, we will review the nanomedicine-based strategies for TME modulation in HCC (Table 1).

### 3.1. Anti-Angiogenesis Nanotherapy

In response to the low level of oxygen, cancer cells promote the angiogenesis of tumors by an imbalance between pro-and anti-angiogenic factors [33]. Through neovascularization, more delivery of oxygen and nutrients promote tumor proliferation [63]. However, the rapid and uncontrolled growth of tumors causes more severe hypoxia thus creating a vicious cycle. Additionally, because of anatomical and functional vascular abnormalities, therapeutic drug delivery are strongly impaired [83]. So, modulating tumor vessels might be a viable approach to increase the effectiveness of tumor treatment.

Anti-angiogenic therapy is widely accepted and used in treating HCC [84]. In fact, most currently approved first- and second-line therapies for advanced HCC target angiogenic pathways, in which the VEGF/VEGF receptor (VEGFR) signaling pathway has been validated as a therapeutic target in HCC [85]. Though sorafenib and Lenvatinib exert anti-angiogenic and antiproliferative effects are the first-line treatment options, the drugs are rarely delivered at high concentrations to reach the cancerous tissues. Nanoparticles, as an effective platform for drug delivery, can overcome the adverse side effects of systemic chemotherapeutic administration by improving their pharmacokinetics and accumulation in tumor sites [86,87].

**Figure 2:**
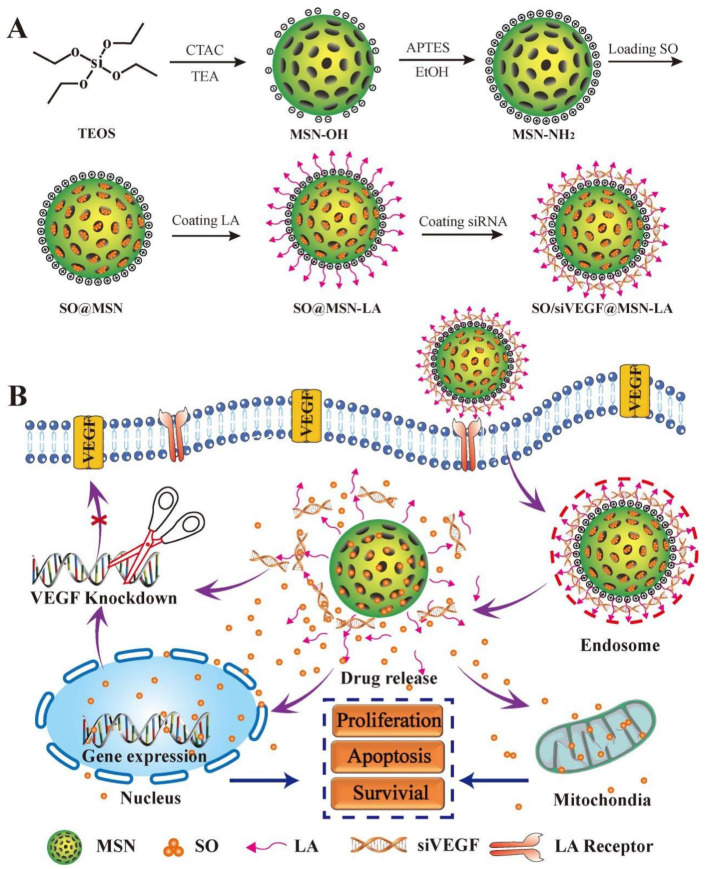
(**A**) Schematic illustration of synthesis procedure of SO/siVEGF@MSN-LA NPs and (**B**) inhibiting effect on the proliferation of cancer cells. MSN: mesoporous silica nanoparticles; SO: sorafenib; LA: lactobionic acid; siVEGF: vascular endothelial growth factor small interfering RNA. (Copyright © 2017 Elsevier B.V. All rights reserved, https://doi.org/10.1016/j.ejps.2017.10.036).

Nanomedicines can be delivered to tumor sites by active and/or passive targeting. In passive targeting, nanovectors are deposed within the TME due to the leaky vasculature and impaired lymphatic drainage [88]. Recently, a nanoassemblie based on biodegradable dendritic polymers poly(amidoamine)-poly(γ-benzyl-L-Glutamate)-b-D-α-tocopheryl polyethylene glycol 1000 succinate (PAM-PBLG-b-TPGS) to carry sorafenib have been developed. Under physiological conditions, the nanoassemblie releases a small portion of sorafenib, which indicates its characteristic stability [89]. Compared with the free sorafenib, the nanoassemblie induces higher therapy efficiency of HCC in both vitro and vivo, which may be attributed to the high accumulation of nanoparticles in HCC. In addition to anti-angiogenic drug delivery, down-regulating the production of VEGF is another nano-therapeutic strategy against angiogenesis in the HCC. Despite the great therapeutic potential of siRNA, the rapid degradation by nucleases and poor internalization by cancer cells restrict their application [90]. Thus, Han, L. et al. developed oral polymeric nanoparticles based on trimethyl chitosan-cysteine (GTC) conjugate to effectively deliver VEGF small interfering RNA (siVEGF) and survivin short hairpin RNA-expression pDNA (iSur-pDNA) [91]. According to the ELISA assay, GTC nanoparticles can effectively silence VEGF with a reduction of 70.2%. Zheng, et al. have developed an ASGPR-targeting nanovector that delivers sorafenib and siVEGF simultaneously to enhance the targeting ability of the nanodrug delivery system and significantly induce cytotoxicity of three different HCC cell lines [92], which showed the high anti-tumor efficiency as a potential nanovector for targeted delivery to HCC (Figure 2).

Aside from VEGF inhibitors, vascular disruption agents (VDAs) are another type of medicine that can electively disrupt established tumor blood vessels causing necrosis in the center of HCC due to a lack of blood supply. As a representative VDA, combretastatin A4-phosphate (CA4P) has entered phase III clinical trials [93]. Wang, Y. et al. designed a pH-sensitive nanoparticle based on N-urocanyl pullulan (URPA) loaded with the anti-angiogenic drug combretastatin A4 (CA4) and cytotoxic drugs methotrexate (MTX) [94]. The experiments demonstrated that CA4/MTX-URPA exhibited significant inhibitory effects on tumor angiogenesis and growth. However, the use of CA4P frequently upregulates VEGF expression, which limits its application [95]. This disadvantage might be addressed when VDAs were combined with VEGF/VEGFR2 inhibitors which can inhibit the activity of VEGF in response to CA4P, momentarily normalizing the tumor vasculature. Bao, X. et al. designed poly (L-glutamic acid)-graft-methoxy poly (ethylene glycol) containing CA4 (CA4-NPs), and investigated the effectiveness of CA4-NPs together with VEGF/VEGFR2 inhibitor DC101 in improving anti-PD-1 therapy in an H22 tumor model [96]. Immunofluorescent images of the tumors showed that CA4-NP + DC101 co-treatment could normalize tumor vasculature, enhance tumor pericyte coverage, enhance tumor blood vessel perfusion, and overcome tumor hypoxia. Meanwhile, combining CA4-NP with DC101 raised the proportion of intra-tumoral CD8^+^ T cells, which significantly improved the treatment efficacy of anti-PD-1 in HCC.

Moreover, nanoparticles can simultaneously deliver anti-angiogenic agents and other drugs to achieve their spatiotemporal cooperation in tumors, improving the efficacy of cancer treatment. Chang et al. developed a tumor-targeted multifunctional nanoparticle MnO_2_ and a shell composed of lipids and poly(lactic-co-glycolic) acid (PLGA) loaded with sorafenib. These multifunctional nanoplatforms co-deliver sorafenib and MnO_2_ for oxygen production to overcome hypoxia-induced drug resistance [97]. Since the favorable drug delivery system is expected to selectively deliver drug payloads in tumor sites and be time-release controlled, different stimulus-responsive nanoparticles, releasing drugs triggered by various external or internal stimuli, are tailored [98]. Zhang et al. designed a pH-sensitive nanoparticle for co-delivering the pro-apoptotic drug DOX and anti-angiogenic drug curcumin [99]. This nanoplatform promoted a spike in drug release in the acid TME. Curcumin inhibits the expression of VEGFR-1, VEGFR-2, VEGFR-3, and epidermal growth factor receptors [100]. Meanwhile, curcumin suppresses the main caspase pathway and activates the main caspase-independent pathway to reduce the adverse effects associated with doxorubicin [101]. Compared with chemotherapy alone, combining treatment with anti-angiogenic medications can enhance the therapeutic efficiency synergistically.

Anti-angiogenic therapy, on the other hand, must address a number of concerns. Firstly, new targets for anti-angiogenic therapy are needed. To date, the majority of anti-angiogenic drugs have targeted VEGF/VEGFR signaling pathways in tumor endothelial cells. However, tumor endothelial cells are heterogeneous [102]. Therefore, more investigation is necessary to explore new targets for anti-angiogenic therapy that increase angiogenesis capability. Moreover, achieving efficient and accurate delivery of nanocarrier to tumor sites remains a stumbling block in nanomedicine. For the application of nanomedicine in HCC, circulation, stability, degradability, and the balance between side effects and curative efficacy must all be carefully studied.

### 3.2. Nanomedicines Designed to Overcome Tumor Physiological Barrier

**Figure 3:**
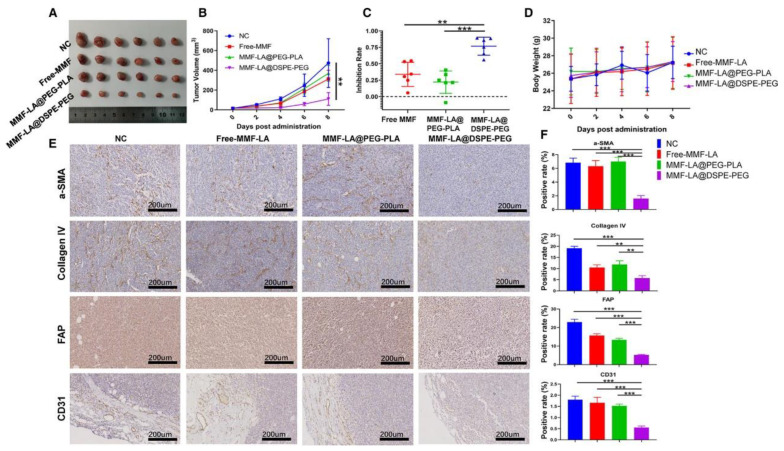
MMF-LA@DSPE-PEG inhibited HCC growth by depleting CAF. Mice were orally administrated with free MMF (20 mg/kg) or intravenously injected with MMF-LA NPs (at 20 mg/kg MMF-equivalent dose) every other day four times. (**A**), Tumor images of different groups, (n = 6). (**B**), Tumor growth curves of different groups, ** *p* < 0.01. (**C**), Tumor inhibition rates of different treatments. (n = 6), ** *p* < 0.01, *** *p* < 0.001. (**D**), Bodyweights (mean ± SD, n = 6) of mice in different groups. (**E**), Expression levels of α-SMA, FAP, collagen IV, and CD31 determined by Immunohistochemistry. The scale bars: 200 µm. (**F**), Quantitative analysis of panel E (Image J software), data are shown as the mean ± SD, (n = 3), ** *p* < 0.01, *** *p* < 0.001. MMF-LA: Mycophenolate mofetil-linoleic acid; DSPE-PEG: 1, 2-distearoyl-sn-glycero-3-phosphoethanolamine-N-poly (ethylene glycol); CAF: cancer-associated fibroblast; α-SMA: alpha-smooth muscle actin; FAP: fibroblast activation protein. (© 2021 The Authors. Journal of Cellular and Molecular Medicine published by Foundation for Cellular and Molecular Medicine and John Wiley & Sons Ltd. https://doi.org/10.1111/jcmm.16434).

Current studies aim to regulate the ECM in two ways: degradation and stiffness. The disruption of the balance between degradation and stiffening is contributed to tumor growth and progression [103]. ECM stiffness can be targeted by reprogramming CAFs and blocking the TGF-β signal pathway [43]. Matrix metalloproteinase (MMP) inhibitors can be used to suppress ECM degradation [104]. For example, Liang, S. et al. constructed a stroma modulation nanosystem based on PEG–PLGA nanospheres [105]. The immunohistochemistry images showed that ECM formation collagen fibers were significantly reduced, via inhibiting TGF-β signaling. The regulation of the tumor ECM greatly enhanced the penetration of nanospheres and facilitated further tumor therapy. HCC is frequently accompanied by marked fibrosis [106]. Mycophenolic acid, the active metabolite of mycophenolate mofetil, exhibits a powerful antifibrotic activity [107]. Yang, Z. et al. designed nanoparticles loaded with mycophenolate mofetil based on 1, 2-distearoyl-sn-glycero-3-phosphoethanolamine-N-poly (ethylene glycol) (MMF-LA@DSPE-PEG) target CAFs [108] (Figure 3). It was shown that the number of CAFs accumulated in tumors was remarkably reduced, as the expression levels of proteins associated with CAF, such as α-smooth muscle actin (α-SMA), fibroblast activation protein (FAP), and collagen IV, were significantly decreased. In mouse models bearing HCC xenograft, mycophenolate mofetil-loaded nanoparticles significantly suppressed fibrotic as well as tumor progression.

In addition to regulating ECM stiffness, some researchers have focused on MMPs as a chemotherapy target in the HCC. MMPs are zinc-dependent endopeptidases that are responsible for degrading basement membrane and various proteins in EMC. According to unambiguous evidence, the release and activation of MMPs facilitate the migration and infiltration of the HCC cells through the damaged basement membrane [109,110]. Moreover, co-workers reported “two-in-one” nanofiber systems containing an anti-tumor drug (DOX) and an MMP inhibitor hexapeptide (KGFRWR) (DOX-KGFRWR) [111]. After administration, the initial liquid DOX-KGFRWR transitioned into nanofibers in the tumor sites, contributing to the inhibition of MPP and antiproliferative effect on HCC. As the results showed, DOX-KGFRWR enhanced the local concentration in the HCC and exerted a synergistic inhibiting effect on HCC cells (SMMC7721) migration. DOX-KGFRWR not only suppressed tumor growth in situ but decreased the number of metastatic nodules. On the other hand, Yeow et al. verified that specific ECM depletion is a viable strategy for boosting the accumulation and uptake of nanoparticles in poorly perfused malignancies such as HCC [112]. According to their results, the lectin-staining in HCC treated with ECM depletion was significantly higher than with PBS, which improved blood vessel function and perfusion in HCC. Notably, nanocarrier itself benefits from ECM depletion therapy. Decreasing the amount of ECM in advance induced significantly higher nanoparticle accumulation in HCC. So, the combination of nanoparticles and ECM depletion might be an ideal option. Based on the above hypothesis, Luo, J. et al. developed a chondroitin sulfate (CSN) modified lipid nanoparticles co-delivery, an ECM depletion drug (retinoic acid, RA), and a chemotherapy drug (DOX) (DOX + RA-CSNs) [113]. The nanoparticle delivery system DOX + RA-CSNs for the Golgi apparatus-specific delivery inhibited the production of type I collagen, which complements the anti-tumor effects of DOX loaded within the nanoparticles. Importantly, the collapse of the ECM barrier greatly boosted the accumulation of DOX + RA-CSNs in HCC and improved the uptake of DOX and RA in HCC cells.

### 3.3. Nanomedicine for Immunosuppressive Cells Inhibition

#### 3.3.1. MDSCs Regulating Nanomedicine

The recruited MDSCs in TME act as a major obstacle for immunotherapy, which plays an important role in immune escape. The following steps have been proposed for therapeutic targeting of MDSCs: (1) interfering with their production by regulation of myelopoiesis, (2) promoting MDSCs differentiation into mature fully mature myeloid cells, (3) eliminating MDSCs, and (4) suppressing their immunosuppressive function.

The chemotherapeutic drug gemcitabine was able to selectively reduce the majority of s MDSCs in tumor-bearing animals while having no effect on macrophages, CD4^+^/CD8^+^ T cells, B cells, or NK cells [114]. To encapsulate Gem derivatives, Suzuki, E. et al. designed a lipid-coated calcium phosphate (LCP) nanocarrier which could effectively deplete MDSCs in the B16F10 mouse melanoma model. Plebanek, M.P. et al. designed high-density lipoprotein-like nanocarriers, with a strong affinity to scavenger receptor type B expressed by MDSCs, to suppress the function of MDSCs [115]. For instance, Lai, C. et al. designed folate (FA) modified chitosan nanoparticles loaded with mouse interferon-γ-inducible protein-10 (mIP-10) plasmid (FA-chitosan/mIP-10) which could efficiently attract and activate T cells, B cells and NK cells with an increase in the number of MDSCs [116] on HCC tumor models. Therefore, Hu, Z. et al. combined FA-chitosan/mIP-10 with DC/tumor fusion vaccine to improve the immunosuppressive TME and enhance anti-cancer efficiency [117]. The results showed that compared with the administration of FA-chitosan/mIP-10 alone, the growth of implanted HCC tumors was effectively inhibited upon the treatment with both FA-chitosan/mIP-10 and DC/tumor fusion vaccine. The results suggested that DC/tumor fusion vaccine, together with FA-chitosan/mIP-10, greatly increased anti-tumor immune responses which inhibited the recruitment of MDSCs. In comparison to other malignancies, however, a few researchers have looked at the regulatory influence of nanoparticles on MDSCs in HCC. At present, MDSCs were generally regulated by combining other immune cell therapy. Further studies targeting MDSCs specifically are necessary due to the importance of MDSCs in HCC progression.

#### 3.3.2. T Cell-Modulating Nanoparticles

T cells are crucial components of the adaptive immune system that help to defend against pathogens like viruses, bacteria, and cancers. T cells are classified into three categories based on their functions: helper T lymphocytes (HTLs), Tregs, and cytotoxic T lymphocytes (CTLs). The presence of a large number of Treg cells in the TME, as well as a low CD8^+^ T cells to Treg cells ratio, is linked to poor prognosis, suggesting that Treg cells block tumor antigen-specific T cell immune responses [118]. Treg cell elimination or modulation of its activities may provide potential immunotherapies. In view of the vital key of T cells in cancer immunotherapy, we look at nanoparticles that control T cell viability in the following section.

**Figure 4:**
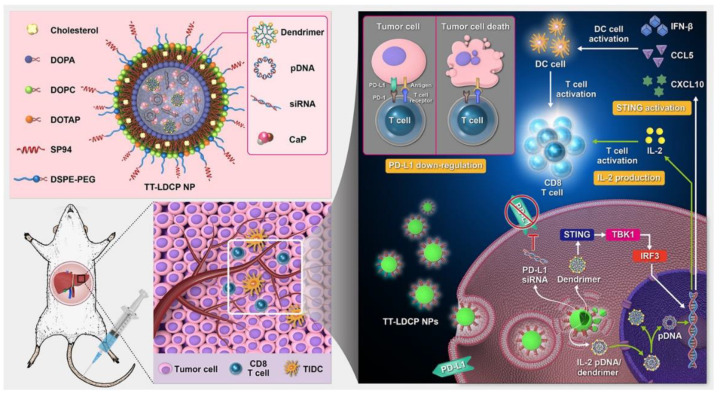
Schematic representation of the mechanism of immunogene therapy by TT-LDCP NPs containing siRNA against the immune checkpoint PD-L1 and Pdna encoding the immunostimulating cytokine IL-2. Active tumor targeting was achieved through the addition of the HCC-targeted SP94 peptide to the surface of the NPs.

IL-2, which is recognized as a T cell growth factor to enhance memory T cell responses and regulate T cell maintenance, is the first FDA-approved immunotherapy for human cancer [119]. Treg cells that express the transcription factor Foxp3 play an important role in immune tolerance and autoimmunity prevention and a low dose of IL-2 has been proven to boost Tregs and improve their suppressive abilities [120]. Tregs consume IL-2 primarily through high-affinity IL-2 receptors (CD25), which limit the amount of IL-2 available for effector T cell proliferation and activation. Therefore, injection of a sufficient dose of IL-2 can neutralize Tregs suppressive abilities. In order to obtain sufficient exposure at tumor sites and induce tumor suppression with decreasing side effects, several investigations have focused on nanocarrier-based IL-2 application. Wu, J. et al. developed an N, N, N-trimethyl chitosan (TMC) based nanocarrier to realize co-delivery DOX and recombinant human IL-2 (FTCD/rhIL-2) which increased the anti-cancer therapeutic benefits with toxicity reduced [121]. These nanoparticles could suppress tumor progression through apoptosis induced by DOX and enhance anti-cancer immunity by rhIL-2. The nanocomplexes FTCD/rhIL-2 could promote humoral and cellular immunity by activating the vitality of T, B lymphocytes, and NK cells. The in vivo investigations in an HCC model have revealed that FTCD/rhIL-2 exhibited stronger anti-tumor efficacy than DOX or rhIL-2, respectively. In addition to administrating IL-2 protein directly, delivery of immunostimulatory IL-2–encoding plasmid DNA (Pdna) can also remodel the immunosuppressive TME of HCC. Huang, K.-W. et al. developed tumor-targeted lipid-dendrimer-calcium-phosphate nanoparticles (TT-LDCP) loaded with siRNA silencing immune checkpoint ligand PD-L1 gene and Pdna upregulating expression of the immunostimulating cytokine IL-2 [122] (Figure 4). Confocal microscopy detection of fluorescence intensity showed that TT-LDCP nanoparticles could efficiently deliver siRNA and Pdna into two HCC cell lines (murine HCA-1 and human Hep3B) with effective gene transfection. Experiments showed that TT-LDCP nanoparticles that co-delivered PD-L1 siRNA and IL-2 Pdna could reverse the immunosuppressive TME of HCC by increasing tumoral infiltration CD8^+^ T cells and promote the maturation of tumor-infiltrating DCs.

The thymine-capped PAMAM dendrimer/CaP complexes achieved highly efficient gene transfection efficacy by enhancing the nuclear delivery of the Pdna. Furthermore, thymine-capped PAMAM dendrimers stimulate the STING pathway and serve as an adjuvant to promote the maturation of intra-tumoral DCs. Efficient tumor-targeted co-delivery of PD-L1 siRNA and IL-2 Pdna achieves tumor-specific expression of IL-2 and down-regulation of PD-L1, increases infiltration and activation of CD8+ T cells in HCC, and induces a strong tumor-suppressive effect in HCC in synergy with a vaccine. CaP, calcium phosphate; TIDC, tumor-infiltrating dendritic cell; TT-LDCP NPs, tumor-targeted lipid-dendrimer-calcium-phosphate NPs; IFN-γ, interferon-γ. (Copyright © 2020 The Authors, some rights reserved; exclusive licensee American Association for the Advancement of Science. http://dx.doi.org/10.1126/sciadv.aax5032).

IL-12 is another potent cytokine in provoking anti-tumor immune responses [123]. Li, J. et al. newly created CD8 and Glypican-3 antibodies modified PLGA nanoparticles loaded with IL-12 [123]. Cell counting revealed that compared with other groups, the proliferation of CD8^+^ T cells was more effective in the group treated with the targeted immune nanoparticles (TINPs). TINPs are attached precisely to the two target cells (CD8^+^ T cells and HepG-2 cells) to form T cell-HepG-2 cell clusters to induce robust immune responses. Moreover, compared to soluble IL-12, the expression of CD107a, which was a degranulation marker and a predictor of T lymphocytes’ ability to lyse tumor cells, was 5-fold higher when treated with TINPs.

The metabolism in tumors differs from the normal tissues from which they develop, indicating that metabolic pathways may make effective targets for cancer therapy [124]. Accelerated glycolysis of a tumor, known as the “Warburg effect”, leads to increased lactate production [125]. Lactate, which drives cancer cells, has been demonstrated to strongly inhibited the activation of T cells [126,127]. 2-Deoxy-D-glucose (2DG), a hexokinase inhibitor, can interrupt glycolysis [128]. Sasaki, K. et al. designed 2DG-encapsulated PLGA nanoparticles (2DG-PLGA-NPs) to improve the delivery efficiency of 2DG to HCC [129]. It was found that 2DG-PLGA-NPs may boost T-cell trafficking in the TME by reducing the generation of lactate by tumor cells and increase the production of IFN-γ and the uptake of glucose by CD8^+^ T cells.

#### 3.3.3. TAM Modulating Nanoparticles

In view of the key role of macrophages in cancer immunity, current therapies targeting TAMs utilize four strategies: (1) restricting macrophage recruitment, (2) depleting TAMs, (3) re-educating TAMs, and (4) blocking the CD47-signal regulatory protein alpha (SIRPα) pathway.

Several studies have proven that hypoxia induced by sorafenib could upregulate the expression of stromal-derived factor 1α (SDF-1α) and its receptor, C-X-C receptor type 4 (CXCR4) in HCC [130,131]. AMD3100, a CXCR4 inhibitor, could suppress cancer cell proliferation and M2-TAM polarization by blocking SDF1α/CXCR4 pathway [130]. Gao, D. et al. contrasted AMD3100 modified lipid-coated PLGA nanoparticles with sorafenib-containing (ADOPSor-NPs) [132]. These ADOPSor-NPs delivered sorafenib and AMD3100 into HCC, triggered tumor apoptosis, prevented the infiltration of TAMs, and overcame the acquired sorafenib resistance. In the orthotopic HCC mice model, ADOPSor-NPs effectively suppressed primary HCC development and metastasis and thus improved overall survival. Li, G. et al. prepared a nanoliposome loaded a sphingolipid metabolite C6-ceramide (LipC6). In liver tumor-bearing mice, LipC6 administration decreased the quantity of TAMs and their capacity to inhibit the anti-tumor immune response [133].

Apart from decreasing TAMs infiltration, another strategy is to re-educate TAMs. In response to changes in the TME during tumor progression, the TAMs go through a shift of polarized phenotypes from M1 to M2. The macrophage, on the other hand, retains the ability for plasticity, including the capacity to transition between M1/M2 status in response to microenvironmental signals. A number of studies have looked into the applications of nanoparticles to modulate TAM polarization from an immune-suppressive phenotype to an immune-promoting one [134]. Wang, T. et al. created twin-like core-shell nanoparticles: SF loaded cationic lipid-based nanoparticles (CLN) coated with *O*-Carboxymethyl-chitosan (CMCS) (CMCS/SF-CLN) and mannose-modified IMD-0354 (a TAM re-polarization agents) loaded CLN coated with CMCS (M-IMD-CLN) [135]. To improve tumor-localized chemoimmunotherapy, CMCS/SF-CLN and CMCS/M-IMD-CLN could simultaneously target cancer cells and TAM separately via SF and mannose on the surface of CLN. Flow cytometry assay showed that the M1/M2 ratio of CMCS/M-IMD-CLN was ~2.5-fold higher than the PBS group, which indicated enhanced polarization. Immunogenic cytokines IFN-γ and IL-12 secreted by M1-TAM were higher than those in CMCS/SF-CLN. Moreover, the administration of CMCS/M-IMD-CLN normalized abnormal tumor blood vessels induced by CMCS/SF-CLN. These findings revealed that CMCS/M-IMD-CLN considerably improved the immunosuppression caused by CMCS/SF-CLN via M2-TAM polarization. In order to deliver siRNA to M2-TAM selectively, Kaps, L. et al. prepared α-mannosyl modified cationic nanohydrogel particles (ManNP) loaded with siRNA [136]. ManNP specifically targeted M2-TAMs with no organ or cellular toxicity, indicting them as promising nanocarriers for macrophage repolarization in HCC.

Hypoxia is frequent in HCC, which leads to an inhibitory TME, such as macrophage recruitment and polarization. In other words, improving hypoxia contributes to a reduction in the amounts of TAMs as well as in transit pro-tumor M2-TAM into anti-tumor M1-TAM [137]. Dai, X. et al. synthesized oxygen microcapsules based on polydopamine nanoparticles to improve the hypoxia microenvironment in HCC [138]. The ratio of TAMs to total lymphocytes in the TME of radiation + oxygen microcapsules group showed a significant drop of 55.8% when compared with the PBS group, indicating the suppressed TAMs recruitment. Meanwhile, radiotherapy combined with oxygen microcapsules reprogramed M2-TAMs towards an M1-type phenotype. In detail, the ratio of M1/M2 in the radiotherapy + oxygen microcapsules group was 33-fold higher than in the PBS group.

The binding of the SPIRα on macrophages to CD47, a “don’t eat me” signal on cancer cells, protects cancer cells from being phagocytosed. Thus, blocking the CD47-SIRPα pathway can enhance the phagocytosis of macrophages. Comparetti, E.J. et al. reported that plasma membrane-derived nanostructures (MNPs), co-delivery siRNA (inactivation of the proto-oncogene c-MYC), and the immune adjuvant monophosphoryl lipid A (MPLA) (MNPs-MPLA-siRNA) [139]. The prepared MNPs-MPLA-siRNA downregulated CD47 and PD-L1 expression on Hep-G2 cells and upregulated expression of classical activation markers on macrophages, such as CD64, CD80, CD83, and CD86 (Table 1).

**Table 1:** Nanomedicine-based strategies for TME modulation in HCC.

Target	NP	Size (nm)	Mechanism	Animal Model	Cell Lines	Ref
Anti-angiogenesis	encapsulating sorafenib with PAM-PBLG-b-TPGS	118.3 ± 5.1	release sorafenib target angiogenic pathways	Balb/C nude mice	HepG2 and LO2	[89]
	galactose modified GTC co-delivery iSur-Pdna and siVEGF	130−160	VEGF was depleted with siVEGF	female Balb/c nude mice and female Kunming mice	BEL-7402	[91]
	co-delivery of sorafenib and siVEGF based on mesoporous silica nanoparticles	148.5 ± 3.5	sustained release of sorafenib and siVEGF	NA	HepG2, Huh, HeLa and A549	[92]
	MTX and CA4 loaded N-urocanyl pullulan	187.1 ± 15.2	Release anti-tumor drug MTX and vascular disruption agents CA4	Balb/c and nude mice	HepG2, PLC/PRF/5 and A549	[94]
ECM/CAF	loaded MMF based on 1, 2-distearoyl-sn-glycero-3-phosphoethanolamine-N-poly	156.23 ± 60.38	MMF inhibited fibroblasts proliferation and tubulin expression; reduced CAF density	C57BL/6 mice, nude mice	Huh7, SUN 449, LM3, LX2, Hep1-6, NIH-3T3	[108]
	DOX-KGFRWR	long nanofibers with average widths of 10.51 nm	MMP inhibition and antiproliferative effects	male Sprague–Dawley rats; male Institute of Cancer Research mice	SMMC7721	[111]
	RA- and DOX-loaded lipid nanoparticles modified with chondroitin sulfate	smaller than 100	RA disrupted the ECM barrier by destroying the Golgi structure of hepatoma cells and HSCs, while DOX-induced cell death.	Male Kunming mice	SMMC-7721 and H22	[113]
MDSC	FA-chitosan/mIP-10 nanoparticles	315.5	sustained local IP-10 expression reduced the number of MDSCs, and attracted CXCR^3+^CD^8+^ T cells to the tumor	Female C57BL/6 mice	Hepa1-6	[117]
T cell	FA modified TMC co-delivery DOX and IL-2	198.1 ± 1.4	improve the amounts of infiltrated cytotoxic T lymphocytes cells.	Female Kunming mice	SMMC-7721 and A549	[121]
	poly(d,l-lactide-co-glycolide) nanoparticle, by loading IL-12 and modifying with CD8 and Glypican-3 antibodies o	145−172	target T cells and deliver IL-12 to T cells for effective activation and proliferation.	NA	HepG-2	[123]
	2DG-encapsulated PLGA nanoparticles	120	activated CD8+ T-cell chemotaxis in the tumor microenvironment via the decreased production of lactate in tumors, the increased IFN-γ production and glucose uptake in CD8+ T cells, and production of CXCL9/CXCL10/CXCL11 in both the tumors and CD8+ T cells	nude mice with xenograft tumors	The Huh7, HepG2, B16F10, BxPC3, OS-RC-2, and HT29 cells	[129]
TAM	AMD3100 modified lipid-coated PLGA nanoparticles with sorafenib-containing	150−200	suppressed the infiltration of TAMs	Male C3H/HeNCrNarl mice	HCA-1 and JHH-7	[132]
	a nanoliposome-loaded C6-ceramide	NA	reduces not only TAM frequency but also its suppressive function and increased the activity of CD8+ T cells	Male C57BL/6 mice	TAg-transformed B6/WT-19 cells	[133]
	mannose-modified IMD-0354 loaded cationic lipid-based nanoparticles coated with polymer O-carboxymethyl-chitosan	129.4 ± 6.8	TAM re-polarization	C57BL/6 mice	Hepa1-6	[135]
	MNPs-MPLA-siRNA	40−400	inhibiting the activity of c-MYC oncogene to reduce the pro-tumoral response from M2 macrophages.	NA	Hep-G2	[139]

PAM-PBLG-b-TPGS: poly(amidoamine)-poly(γ-benzyl-L-Glutamate)-b-D-α-tocopheryl polyethylene glycol 1000 succinate; GTC: trimethyl chitosan-cysteine; VEGF: vascular endothelial growth factor; NA: not available; MTX: methotrexate; CA4: combretastatin A4; ECM: extracellular matrix; CAF: cancer-associated fibroblasts; MMF: mycophenolate mofetil; DOX-KGFRWR: doxorubicin-conjugated hexapeptide; MMP: matrix metalloproteinases; RA: retinoic acid; HSCs: Hepatic stellate cells; MDSC: Myeloid-derived suppressor cells; FA: folate; mIP-10: mouse interferon-induced protein-10 gene; TMC: N,N,N-trimethyl chitosan; IL-2: Interleukin-2; 2DG: 2-deoxy-D-glucose; PLGA: poly(lactic-co-glycolic acid); IFN-γ: Interferon-γ; TAM: tumor-associated macrophage; MNPs: Plasma membrane-derived nanoparticles co-delivery monophosphoryl lipid A and small interfering RNA.

## 4. Conclusions and Future Perspectives

HCC is one of the most prevalent malignancies in the world, with rising incidence and high mortality rates. Immunotherapy for HCC is both promising and challenging due to its unique characteristic of immunity and immune tolerance. As a protective “ecological niche” for tumor cells, the different components and complex crosstalk in TME promote HCC progression and impair therapeutic effects. Since the TME of HCC plays a key role in its initiation and progression, it is worth considering the regulation of TME to enhance anti-cancer immune responses. Given the rapid development of nanotechnology and the success of cancer immunotherapy in the clinic, the convergence of the two therapies will certainly achieve significant progress in cancer treatment. In this study, we review recent advancements in the treatment of HCC using nano-delivery technologies to regulate immunosuppressive TME. There is a plethora of studies to reprogram the components of TME, such as tumor cells, T lymphocytes, tumor endothelial cells, TAMs, and ECM. TME-modulating nanoparticles can contain various drugs and be modified by targeting ligands in order to highly and specifically accumulate in tumor sites while reducing side effects.

However, there exist a few obstacles to be faced and overcome when it comes to regulating the HCC microenvironment. For example, despite the fact that TME-modulating nanoparticles have demonstrated promising results in preclinical studies, several challenges remain in their clinical translation. First of all, the potential toxicity and immunogenicity of nanomaterials restrict their application in clinical experiments. Immune responses toward the nanomaterials may induce severe complications, such as allergic reactions, thrombogenesis, and so on [140]. Thus, future clinical translations of nanoparticles should concentrate on the low antigenicity with a carefully controlled dose. Secondly, considering the unique immunological landscape of HCC, which contains large amounts of immune cells and some of these, such as Kupffer cells, cannot be found in any other parts of the body, the components in the HCC microenvironment should be further investigated. The TME is a complex network and the impact of one component’s depletion or suppression on the entire system is unknown. Inhibition of one or more components in HCC may be compensated by overexpression of other pathways. A better understanding of components in TME of HCC and the long-term effects of nanoparticles targeting these TME components is critical in future research. Thirdly, because of the existing individual differences in reactions to nanomedicines, it will also be important to develop biomarkers that are both reliable and predictive.

Altogether, modulation of the TME of HCC is seen to be promising as it can effectively improve anti-cancer immunity. Significant progress in the treatment of HCC is believed to be made in the near future.

## Data Availability

Not applicable.

## Abbreviations

HCC: hepatocellular carcinoma; TME: tumor microenvironment; FDA: Food and Drug Administration; DC: dendritic cells; ECM: extracellular matrix; EPR: the enhanced permeability and retention; TAM: tumor-associated macrophages; MDSC: myeloid-derived suppressor cells; VEGF: vascular endothelial growth factor; bFGF: basic fibroblast growth factor; IL-8: interleukin-8; CAFs: cancer-associated fibroblasts; PMN-MDSC: polymorphonuclear-MDSC; M-MDSC: monocytic MDSC; HBV: hepatitis B virus; Tregs: regulatory T cells; TGFβ: transforming growth factor-β; VEGFR: VEGF receptor; PAM-PBLG-b-TPGS: poly(amidoamine)-poly(γ-benzyl-L-Glutamate)-b-D-α-tocopheryl polyethylene glycol 1000 succinate; GTC: trimethyl chitosan-cysteine; siVEGF: VEGF small interfering RNA; iSur-pDNA: survivin hort hairpin RNA-expression pDNA; ASGPR: asialoglycoprotein receptor; VDAs: vascular disruption agents; CA4P: combretastatin A4-phosphate; URPA: N-urocanyl pullulan; MTX: methotrexate; PLGA: poly(lactic-co-glycolic) acid; MMP: matrix metalloproteinase; DOX: doxorubicin; CSN: chondroitin sulfate; LCP: lipid-coated calcium phosphate; mIP-10: mouse interferon-γ-inducible protein-10; FA: folate; HTLs: helper T lymphocytes; CTLs: cytotoxic T lymphocytes; TMC: N, N, N-trimethyl chitosan; rhIL-2: recombinant human IL-2;pDNA: plasmid DNA; TT-LDCP: tumor-targeted lipid-dendrimer-calcium-phosphate; TINPs: targeted immune nanoparticles; SDF-1α: stromal-derived factor 1α; CXCR4: C-X-C receptor type 4; CLN: cationic lipid-based nanoparticles; CMCS: *O*-Carboxymethyl-chitosan; MNPs: plasma membrane-derived nanostructures; MPLA: monophosphoryl lipid A; MMF-LA: Mycophenolate mofetil-linoleic acid; DSPE-PEG: 1, 2-distearoyl-sn-glycero-3-phosphoethanolamine-N-poly (ethylene glycol); α-SMA: alpha-smooth muscle actin; FAP: fibroblast activation protein.

## References

[1] Sung H., Ferlay J., Siegel R.L., Laversanne M., Soerjomataram I., Jemal A., Bray F. (2021). Global Cancer Statistics 2020: GLOBOCAN Estimates of Incidence and Mortality Worldwide for 36 Cancers in 185 Countries. CA Cancer J. Clin..

[2] Llovet J.M., Kelley R.K., Villanueva A., Singal A.G., Pikarsky E., Roayaie S., Lencioni R., Koike K., Zucman-Rossi J., Finn R.S. (2021). Hepatocellular carcinoma. Nat. Rev. Dis. Primers.

[3] Pinato D.J., Sharma R., Allara E., Yen C., Arizumi T., Kubota K., Bettinger D., Jang J.W., Smirne C., Kim Y.W. (2017). The ALBI grade provides objective hepatic reserve estimation across each BCLC stage of hepatocellular carcinoma. J. Hepatol..

[4] Roayaie S., Obeidat K., Sposito C., Mariani L., Bhoori S., Pellegrinelli A., Labow D., Llovet J.M., Schwartz M., Mazzaferro V. (2013). Resection of hepatocellular cancer ≤2 cm: Results from two Western centers. Hepatology.

[5] Roxburgh P., Evans T.R.J. (2008). Systemic therapy of hepatocellular carcinoma: Are we making progress?. Adv. Ther..

[6] Jin H., Qin S., He J., Xiao J., Li Q., Mao Y., Zhao L. (2022). New insights into checkpoint inhibitor immunotherapy and its combined therapies in hepatocellular carcinoma: From mechanisms to clinical trials. Int. J. Biol. Sci..

[7] Al-Salama Z.T., Syed Y.Y., Scott L.J. (2019). Lenvatinib: A Review in Hepatocellular Carcinoma. Drugs.

[8] Thomas M. (2009). Molecular targeted therapy for hepatocellular carcinoma. J. Gastroenterol..

[9] Tang W., Chen Z., Zhang W., Cheng Y., Zhang B., Wu F., Wang Q., Wang S., Rong D., Reiter F.P. (2020). The mechanisms of sorafenib resistance in hepatocellular carcinoma: Theoretical basis and therapeutic aspects. Signal Transduct. Target. Ther..

[10] Van den Bulk J., Verdegaal E.M., de Miranda N.F. (2018). Cancer immunotherapy: Broadening the scope of targetable tumours. Open Biol..

[11] Hoos A., Britten C. (2012). The immuno-oncology framework: Enabling a new era of cancer therapy. Oncoimmunology.

[12] Marrero J.A., Kulik L.M., Sirlin C.B., Zhu A.X., Finn R.S., Abecassis M.M., Roberts L.R., Heimbach J.K. (2018). Diagnosis, Staging, and Management of Hepatocellular Carcinoma: 2018 Practice Guidance by the American Association for the Study of Liver Diseases. Hepatology.

[13] Yau T., Hsu C., Kim T.Y., Choo S.P., Kang Y.K., Hou M.M., Numata K., Yeo W., Chopra A., Ikeda M. (2019). Nivolumab in advanced hepatocellular carcinoma: Sorafenib-experienced Asian cohort analysis. J. Hepatol..

[14] Pan C., Liu H., Robins E., Song W., Liu D., Li Z., Zheng L. (2020). Next-generation immuno-oncology agents: Current momentum shifts in cancer immunotherapy. J. Hematol. Oncol..

[15] Yan Y., Zheng L., Du Q., Yan B., Geller D.A. (2020). Interferon regulatory factor 1 (IRF-1) and IRF-2 regulate PD-L1 expression in hepatocellular carcinoma (HCC) cells. Cancer Immunol. Immunother. CII.

[16] Sharma P., Hu-Lieskovan S., Wargo J.A., Ribas A. (2017). Primary, Adaptive, and Acquired Resistance to Cancer Immunotherapy. Cell.

[17] El-Khoueiry A.B., Sangro B., Yau T., Crocenzi T.S., Kudo M., Hsu C., Kim T.-Y., Choo S.-P., Trojan J., Welling T.H.R. (2017). Nivolumab in patients with advanced hepatocellular carcinoma (CheckMate 040): An open-label, non-comparative, phase 1/2 dose escalation and expansion trial. Lancet.

[18] Sheng H., Huang Y., Xiao Y., Zhu Z., Shen M., Zhou P., Guo Z., Wang J., Wang H., Dai W. (2020). ATR inhibitor AZD6738 enhances the antitumor activity of radiotherapy and immune checkpoint inhibitors by potentiating the tumor immune microenvironment in hepatocellular carcinoma. J. Immunother. Cancer.

[19] Wu Y., Kuang D.M., Pan W.D., Wan Y.L., Lao X.M., Wang D., Li X.F., Zheng L. (2013). Monocyte/macrophage-elicited natural killer cell dysfunction in hepatocellular carcinoma is mediated by CD48/2B4 interactions. Hepatology.

[20] Hato T., Goyal L., Greten T.F., Duda D.G., Zhu A.X. (2014). Immune checkpoint blockade in hepatocellular carcinoma: Current progress and future directions. Hepatology.

[21] Quail D.F., Joyce J.A. (2013). Microenvironmental regulation of tumor progression and metastasis. Nat. Med..

[22] Eggert T., Greten T.F. (2017). Tumor regulation of the tissue environment in the liver. Pharm. Ther..

[23] Sun K., Yu J., Hu J., Chen J., Song J., Chen Z., Cai Z., Lu Z., Zhang L., Wang Z. (2022). Salicylic acid-based hypoxia-responsive chemodynamic nanomedicines boost antitumor immunotherapy by modulating immunosuppressive tumor microenvironment. Acta Biomater..

[24] Dai Q., Wilhelm S., Ding D., Syed A.M., Sindhwani S., Zhang Y., Chen Y.Y., MacMillan P., Chan W.C.W. (2018). Quantifying the Ligand-Coated Nanoparticle Delivery to Cancer Cells in Solid Tumors. ACS Nano.

[25] Golombek S.K., May J.-N., Theek B., Appold L., Drude N., Kiessling F., Lammers T. (2018). Tumor targeting via EPR: Strategies to enhance patient responses. Adv. Drug Deliv. Rev..

[26] Zhao P., Wang Y., Kang X., Wu A., Yin W., Tang Y., Wang J., Zhang M., Duan Y., Huang Y. (2018). Dual-targeting biomimetic delivery for anti-glioma activity via remodeling the tumor microenvironment and directing macrophage-mediated immunotherapy. Chem. Sci..

[27] Wan J.L., Wang B., Wu M.L., Li J., Gong R.M., Song L.N., Zhang H.S., Zhu G.Q., Chen S.P., Cai J.L. (2022). MTDH antisense oligonucleotides reshape the immunosuppressive tumor microenvironment to sensitize Hepatocellular Carcinoma to immune checkpoint blockade therapy. Cancer Lett..

[28] Wang Y., Wang Z., Jia F., Xu Q., Shu Z., Deng J., Li A., Yu M., Yu Z. (2022). CXCR4-guided liposomes regulating hypoxic and immunosuppressive microenvironment for sorafenib-resistant tumor treatment. Bioact. Mater..

[29] Xiao Y., Chen J., Zhou H., Zeng X., Ruan Z., Pu Z., Jiang X., Matsui A., Zhu L., Amoozgar Z. (2022). Combining p53 mRNA nanotherapy with immune checkpoint blockade reprograms the immune microenvironment for effective cancer therapy. Nat. Commun..

[30] Zhang J., Shan W.F., Jin T.T., Wu G.Q., Xiong X.X., Jin H.Y., Zhu S.M. (2014). Propofol exerts anti-hepatocellular carcinoma by microvesicle-mediated transfer of miR-142-3p from macrophage to cancer cells. J. Transl. Med..

[31] Bejarano L., Jordāo M.J.C., Joyce J.A. (2021). Therapeutic Targeting of the Tumor Microenvironment. Cancer Discov..

[32] Petitprez F., Meylan M., de Reyniès A., Sautès-Fridman C., Fridman W.H. (2020). The Tumor Microenvironment in the Response to Immune Checkpoint Blockade Therapies. Front. Immunol..

[33] Jain R.K. (2013). Normalizing tumor microenvironment to treat cancer: Bench to bedside to biomarkers. J. Clin. Oncol..

[34] Wilson W.R., Hay M.P. (2011). Targeting hypoxia in cancer therapy. Nat. Rev. Cancer.

[35] Facciabene A., Peng X., Hagemann I.S., Balint K., Barchetti A., Wang L.-P., Gimotty P.A., Gilks C.B., Lal P., Zhang L. (2011). Tumour hypoxia promotes tolerance and angiogenesis via CCL28 and Treg cells. Nature.

[36] Carmeliet P., Jain R.K. (2011). Principles and mechanisms of vessel normalization for cancer and other angiogenic diseases. Nat. Rev. Drug Discov..

[37] Schaaf M.B., Garg A.D., Agostinis P. (2018). Defining the role of the tumor vasculature in antitumor immunity and immunotherapy. Cell Death Dis..

[38] Poon R.T.-P., Fan S.-T., Wong J. (2001). Clinical Implications of Circulating Angiogenic Factors in Cancer Patients. J. Clin. Oncol..

[39] Kalluri R. (2016). The biology and function of fibroblasts in cancer. Nat. Rev. Cancer.

[40] Yin Z., Dong C., Jiang K., Xu Z., Li R., Guo K., Shao S., Wang L. (2019). Heterogeneity of cancer-associated fibroblasts and roles in the progression, prognosis, and therapy of hepatocellular carcinoma. J. Hematol. Oncol..

[41] Kubo N., Araki K., Kuwano H., Shirabe K. (2016). Cancer-associated fibroblasts in hepatocellular carcinoma. World J. Gastroenterol..

[42] Fu R., Zhang Y.-W., Li H.-M., Lv W.-C., Zhao L., Guo Q.-L., Lu T., Weiss S.J., Li Z.-Y., Wu Z.-Q. (2018). LW106, a novel indoleamine 2,3-dioxygenase 1 inhibitor, suppresses tumour progression by limiting stroma-immune crosstalk and cancer stem cell enrichment in tumour micro-environment. Br. J. Pharm..

[43] Najafi M., Farhood B., Mortezaee K. (2019). Extracellular matrix (ECM) stiffness and degradation as cancer drivers. J. Cell. Biochem..

[44] Carloni V., Luong T.V., Rombouts K. (2014). Hepatic stellate cells and extracellular matrix in hepatocellular carcinoma: More complicated than ever. Liver Int..

[45] Kumar V., Patel S., Tcyganov E., Gabrilovich D.I. (2016). The Nature of Myeloid-Derived Suppressor Cells in the Tumor Microenvironment. Trends Immunol..

[46] Hao X., Sun G., Zhang Y., Kong X., Rong D., Song J., Tang W., Wang X. (2021). Targeting Immune Cells in the Tumor Microenvironment of HCC: New Opportunities and Challenges. Front. Cell Dev. Biol..

[47] Venook A.P., Papandreou C., Furuse J., de Guevara L.L. (2010). The incidence and epidemiology of hepatocellular carcinoma: A global and regional perspective. Oncologist.

[48] Pallett L.J., Gill U.S., Quaglia A., Sinclair L.V., Jover-Cobos M., Schurich A., Singh K.P., Thomas N., Das A., Chen A. (2015). Metabolic regulation of hepatitis B immunopathology by myeloid-derived suppressor cells. Nat. Med..

[49] Zhang X., Fu X., Li T., Yan H. (2019). The prognostic value of myeloid derived suppressor cell level in hepatocellular carcinoma: A systematic review and meta-analysis. PLoS ONE.

[50] Rodríguez P.C., Ochoa A.C. (2008). Arginine regulation by myeloid derived suppressor cells and tolerance in cancer: Mechanisms and therapeutic perspectives. Immunol. Rev..

[51] Wang Y., Zhang T., Sun M., Ji X., Xie M., Huang W., Xia L. (2021). Therapeutic Values of Myeloid-Derived Suppressor Cells in Hepatocellular Carcinoma: Facts and Hopes. Cancers.

[52] Sakaguchi S., Yamaguchi T., Nomura T., Ono M. (2008). Regulatory T Cells and Immune Tolerance. Cell.

[53] Eisenstein E.M., Williams C.B. (2009). The Treg/Th17 Cell Balance: A New Paradigm for Autoimmunity. Pediatr. Res..

[54] Fu J., Xu D., Liu Z., Shi M., Zhao P., Fu B., Zhang Z., Yang H., Zhang H., Zhou C. (2007). Increased regulatory T cells correlate with CD8 T-cell impairment and poor survival in hepatocellular carcinoma patients. Gastroenterology.

[55] Vignali D.A.A., Collison L.W., Workman C.J. (2008). How regulatory T cells work. Nat. Rev. Immunol..

[56] Murray P.J., Allen J.E., Biswas S.K., Fisher E.A., Gilroy D.W., Goerdt S., Gordon S., Hamilton J.A., Ivashkiv L.B., Lawrence T. (2014). Macrophage activation and polarization: Nomenclature and experimental guidelines. Immunity.

[57] Cassetta L., Pollard J.W. (2020). Tumor-associated macrophages. Curr. Biol..

[58] Yang J., Zhang J.-X., Wang H., Wang G.-L., Hu Q.-G., Zheng Q.-C. (2012). Hepatocellular carcinoma and macrophage interaction induced tumor immunosuppression via Treg requires TLR4 signaling. World J. Gastroenterol..

[59] Ju C., Tacke F. (2016). Hepatic macrophages in homeostasis and liver diseases: From pathogenesis to novel therapeutic strategies. Cell Mol. Immunol..

[60] Yin C., Han Q., Xu D., Zheng B., Zhao X., Zhang J. (2019). SALL4-mediated upregulation of exosomal miR-146a-5p drives T-cell exhaustion by M2 tumor-associated macrophages in HCC. Oncoimmunology.

[61] Hou P.-p., Luo L.-j., Chen H.-z., Chen Q.-t., Bian X.-l., Wu S.-f., Zhou J.-x., Zhao W.-x., Liu J.-m., Wang X.-m. (2020). Ectosomal PKM2 Promotes HCC by Inducing Macrophage Differentiation and Remodeling the Tumor Microenvironment. Mol. Cell.

[62] Chen J., Lin Z., Liu L., Zhang R., Geng Y., Fan M., Zhu W., Lu M., Lu L., Jia H. (2021). GOLM1 exacerbates CD8(+) T cell suppression in hepatocellular carcinoma by promoting exosomal PD-L1 transport into tumor-associated macrophages. Signal Transduct. Target. Ther..

[63] LaGory E.L., Giaccia A.J. (2016). The ever-expanding role of HIF in tumour and stromal biology. Nat. Cell Biol..

[64] Dysthe M., Parihar R. (2020). Myeloid-Derived Suppressor Cells in the Tumor Microenvironment. Adv. Exp. Med. Biol..

[65] Cho H., Seo Y., Loke K.M., Kim S.W., Oh S.M., Kim J.H., Soh J., Kim H.S., Lee H., Kim J. (2018). Cancer-Stimulated CAFs Enhance Monocyte Differentiation and Protumoral TAM Activation via IL6 and GM-CSF Secretion. Clin. Cancer Res. Off. J. Am. Assoc. Cancer Res..

[66] Ranganathan R., Madanmohan S., Kesavan A., Baskar G., Krishnamoorthy Y.R., Santosham R., Ponraju D., Rayala S.K., Venkatraman G. (2012). Nanomedicine: Towards development of patient-friendly drug-delivery systems for oncological applications. Int. J. Nanomed..

[67] Turato C., Balasso A., Carloni V., Tiribelli C., Mastrotto F., Mazzocca A., Pontisso P. (2017). New molecular targets for functionalized nanosized drug delivery systems in personalized therapy for hepatocellular carcinoma. J. Control. Release Off. J. Control. Release Soc..

[68] Martin D.N., Uprichard S.L. (2013). Identification of transferrin receptor 1 as a hepatitis C virus entry factor. Proc. Natl. Acad. Sci. USA.

[69] Pramanik A., Laha D., Pramanik P., Karmakar P. (2014). A novel drug “copper acetylacetonate” loaded in folic acid-tagged chitosan nanoparticle for efficient cancer cell targeting. J. Drug Target..

[70] Wang J., Qian Y., Xu L., Shao Y., Zhang H., Shi F., Chen J., Cui S., Chen X., Zhu D. (2020). Hyaluronic acid-shelled, peptide drug conjugate-cored nanomedicine for the treatment of hepatocellular carcinoma. Mater. Sci. Eng. C.

[71] Maghsoudinia F., Tavakoli M.B., Samani R.K., Hejazi S.H., Sobhani T., Mehradnia F., Mehrgardi M.A. (2021). Folic acid-functionalized gadolinium-loaded phase transition nanodroplets for dual-modal ultrasound/magnetic resonance imaging of hepatocellular carcinoma. Talanta.

[72] Zhan X., Guan Y.-Q. (2015). Design of magnetic nanoparticles for hepatocellular carcinoma treatment using the control mechanisms of the cell internal nucleus and external membrane. J. Mater. Chem. B.

[73] Kumari P., Ghosh B., Biswas S. (2016). Nanocarriers for cancer-targeted drug delivery. J. Drug Target..

[74] Shafei A., El-Bakly W., Sobhy A., Wagdy O., Reda A., Aboelenin O., Marzouk A., El Habak K., Mostafa R., Ali M.A. (2017). A review on the efficacy and toxicity of different doxorubicin nanoparticles for targeted therapy in metastatic breast cancer. Biomed. Pharmacother..

[75] Pramanik A., Xu Z., Shamsuddin S.H., Khaled Y.S., Ingram N., Maisey T., Tomlinson D., Coletta P.L., Jayne D., Hughes T.A. (2022). Affimer Tagged Cubosomes: Targeting of Carcinoembryonic Antigen Expressing Colorectal Cancer Cells Using In Vitro and In Vivo Models. ACS Appl. Mater. Interfaces.

[76] Wang Q., Sun Y., Zhang Z., Duan Y. (2015). Targeted polymeric therapeutic nanoparticles: Design and interactions with hepatocellular carcinoma. Biomaterials.

[77] Lou J., Zhang L., Zheng G. (2019). Advancing Cancer Immunotherapies with Nanotechnology. Adv. Ther..

[78] Wu S., Zhang D., Yu J., Dou J., Li X., Mu M., Liang P. (2020). Chemotherapeutic Nanoparticle-Based Liposomes Enhance the Efficiency of Mild Microwave Ablation in Hepatocellular Carcinoma Therapy. Front. Pharmacol..

[79] Cen D., Ge Q., Xie C., Zheng Q., Guo J., Zhang Y., Wang Y., Li X., Gu Z., Cai X. (2021). ZnS@BSA Nanoclusters Potentiate Efficacy of Cancer Immunotherapy. Adv. Mater..

[80] Liao J., Jia Y., Wu Y., Shi K., Yang D., Li P., Qian Z. (2020). Physical-, chemical-, and biological-responsive nanomedicine for cancer therapy. WIREs Nanomed. Nanobiotechnol..

[81] Barenholz Y. (2012). Doxil^®^—The first FDA-approved nano-drug: Lessons learned. J. Control. Release.

[82] Shi J., Kantoff P.W., Wooster R., Farokhzad O.C. (2017). Cancer nanomedicine: Progress, challenges and opportunities. Nat. Rev. Cancer.

[83] Azzi S., Hebda J.K., Gavard J. (2013). Vascular permeability and drug delivery in cancers. Front. Oncol..

[84] Berretta M., Rinaldi L., Di Benedetto F., Lleshi A., De Re V., Facchini G., De Paoli P., Di Francia R. (2016). Angiogenesis Inhibitors for the Treatment of Hepatocellular Carcinoma. Front. Pharmacol..

[85] Taketomi A. (2016). Clinical trials of antiangiogenic therapy for hepatocellular carcinoma. Int. J. Clin. Oncol..

[86] Zhang Y.N., Poon W., Tavares A.J., McGilvray I.D., Chan W.C.W. (2016). Nanoparticle-liver interactions: Cellular uptake and hepatobiliary elimination. J. Control. Release Off. J. Control. Release Soc..

[87] Hao Q., Wang Z., Zhao W., Wen L., Wang W., Lu S., Xing D., Zhan M., Hu X. (2020). Dual-Responsive Polyprodrug Nanoparticles with Cascade-Enhanced Magnetic Resonance Signals for Deep-Penetration Drug Release in Tumor Therapy. ACS Appl. Mater. Interfaces.

[88] Bazak R., Houri M., Achy S.E., Hussein W., Refaat T. (2014). Passive targeting of nanoparticles to cancer: A comprehensive review of the literature. Mol. Clin. Oncol..

[89] Li Z., Ye L., Liu J., Lian D., Li X. (2020). Sorafenib-Loaded Nanoparticles Based on Biodegradable Dendritic Polymers for Enhanced Therapy of Hepatocellular Carcinoma. Int. J. Nanomed..

[90] Kanasty R., Dorkin J.R., Vegas A., Anderson D. (2013). Delivery materials for siRNA therapeutics. Nat. Mater..

[91] Han L., Tang C., Yin C. (2014). Oral delivery of shRNA and siRNA via multifunctional polymeric nanoparticles for synergistic cancer therapy. Biomaterials.

[92] Zheng G., Zhao R., Xu A., Shen Z., Chen X., Shao J. (2018). Co-delivery of sorafenib and siVEGF based on mesoporous silica nanoparticles for ASGPR mediated targeted HCC therapy. Eur. J. Pharm. Sci. Off. J. Eur. Fed. Pharm. Sci..

[93] Chase D.M., Chaplin D.J., Monk B.J. (2017). The development and use of vascular targeted therapy in ovarian cancer. Gynecol. Oncol..

[94] Wang Y., Chen H., Liu Y., Wu J., Zhou P., Wang Y., Li R., Yang X., Zhang N. (2013). pH-sensitive pullulan-based nanoparticle carrier of methotrexate and combretastatin A4 for the combination therapy against hepatocellular carcinoma. Biomaterials.

[95] Inglis D.J., Lavranos T.C., Beaumont D.M., Leske A.F., Brown C.K., Hall A.J., Kremmidiotis G. (2014). The vascular disrupting agent BNC105 potentiates the efficacy of VEGF and mTOR inhibitors in renal and breast cancer. Cancer Biol. Ther..

[96] Bao X., Shen N., Lou Y., Yu H., Wang Y., Liu L., Tang Z., Chen X. (2021). Enhanced anti-PD-1 therapy in hepatocellular carcinoma by tumor vascular disruption and normalization dependent on combretastatin A4 nanoparticles and DC101. Theranostics.

[97] Chang C.-C., Dinh T.K., Lee Y.-A., Wang F.-N., Sung Y.-C., Yu P.-L., Chiu S.-C., Shih Y.-C., Wu C.-Y., Huang Y.-D. (2020). Nanoparticle Delivery of MnO_2_ and Antiangiogenic Therapy to Overcome Hypoxia-Driven Tumor Escape and Suppress Hepatocellular Carcinoma. ACS Appl. Mater. Interfaces.

[98] Karimi M., Ghasemi A., Sahandi Zangabad P., Rahighi R., Moosavi Basri S.M., Mirshekari H., Amiri M., Shafaei Pishabad Z., Aslani A., Bozorgomid M. (2016). Smart micro/nanoparticles in stimulus-responsive drug/gene delivery systems. Chem. Soc. Rev..

[99] Zhang J., Li J., Shi Z., Yang Y., Xie X., Lee S.M., Wang Y., Leong K.W., Chen M. (2017). pH-sensitive polymeric nanoparticles for co-delivery of doxorubicin and curcumin to treat cancer via enhanced pro-apoptotic and anti-angiogenic activities. Acta Biomater..

[100] Arbiser J.L., Klauber N., Rohan R., van Leeuwen R., Huang M.T., Fisher C., Flynn E., Byers H.R. (1998). Curcumin is an in vivo inhibitor of angiogenesis. Mol. Med..

[101] Sadzuka Y., Nagamine M., Toyooka T., Ibuki Y., Sonobe T. (2012). Beneficial effects of curcumin on antitumor activity and adverse reactions of doxorubicin. Int. J. Pharm..

[102] Xiong Y.-Q., Sun H.-C., Zhang W., Zhu X.-D., Zhuang P.-Y., Zhang J.-B., Wang L., Wu W.-z., Qin L.-X., Tang Z.-Y. (2009). Human Hepatocellular Carcinoma Tumor–derived Endothelial Cells Manifest Increased Angiogenesis Capability and Drug Resistance Compared with Normal Endothelial Cells. Clin. Cancer Res..

[103] Chaudhuri O., Koshy S.T., Branco da Cunha C., Shin J.W., Verbeke C.S., Allison K.H., Mooney D.J. (2014). Extracellular matrix stiffness and composition jointly regulate the induction of malignant phenotypes in mammary epithelium. Nat. Mater..

[104] Bourboulia D., Stetler-Stevenson W.G. (2010). Matrix metalloproteinases (MMPs) and tissue inhibitors of metalloproteinases (TIMPs): Positive and negative regulators in tumor cell adhesion. Semin. Cancer Biol..

[105] Liang S., Hu J., Xie Y., Zhou Q., Zhu Y., Yang X. (2016). A polyethylenimine-modified carboxyl-poly(styrene/acrylamide) copolymer nanosphere for co-delivering of CpG and TGF-β receptor I inhibitor with remarkable additive tumor regression effect against liver cancer in mice. Int. J. Nanomed..

[106] Affo S., Yu L.-X., Schwabe R.F. (2017). The Role of Cancer-Associated Fibroblasts and Fibrosis in Liver Cancer. Annu. Rev. Pathol..

[107] Greupink R., Bakker H.I., Reker-Smit C., van Loenen-Weemaes A.M., Kok R.J., Meijer D.K., Beljaars L., Poelstra K. (2005). Studies on the targeted delivery of the antifibrogenic compound mycophenolic acid to the hepatic stellate cell. J. Hepatol..

[108] Yang Z., Zhang L., Zhu H., Zhou K., Wang H., Wang Y., Su R., Guo D., Zhou L., Xu X. (2021). Nanoparticle formulation of mycophenolate mofetil achieves enhanced efficacy against hepatocellular carcinoma by targeting tumour-associated fibroblast. J. Cell Mol. Med..

[109] Arii S., Mise M., Harada T., Furutani M., Ishigami S., Niwano M., Mizumoto M., Fukumoto M., Imamura M. (1996). Overexpression of matrix metalloproteinase 9 gene in hepatocellular carcinoma with invasive potential. Hepatology.

[110] Cui N., Hu M., Khalil R.A. (2017). Biochemical and Biological Attributes of Matrix Metalloproteinases. Prog. Mol. Biol. Transl. Sci..

[111] Ji Y., Xiao Y., Xu L., He J., Qian C., Li W., Wu L., Chen R., Wang J., Hu R. (2018). Drug-Bearing Supramolecular MMP Inhibitor Nanofibers for Inhibition of Metastasis and Growth of Liver Cancer. Adv. Sci..

[112] Yeow Y.L., Wu J., Wang X., Winteringham L., Feindel K.W., Tirnitz-Parker J.E.E., Leedman P.J., Ganss R., Hamzah J. (2022). ECM Depletion Is Required to Improve the Intratumoral Uptake of Iron Oxide Nanoparticles in Poorly Perfused Hepatocellular Carcinoma. Front. Oncol..

[113] Luo J., Gong T., Ma L. (2020). Chondroitin-modified lipid nanoparticles target the Golgi to degrade extracellular matrix for liver cancer management. Carbohydr. Polym..

[114] Suzuki E., Kapoor V., Jassar A.S., Kaiser L.R., Albelda S.M. (2005). Gemcitabine Selectively Eliminates Splenic Gr-1+/CD11b+ Myeloid Suppressor Cells in Tumor-Bearing Animals and Enhances Antitumor Immune Activity. Clin. Cancer Res..

[115] Plebanek M.P., Bhaumik D., Bryce P.J., Thaxton C.S. (2018). Scavenger Receptor Type B1 and Lipoprotein Nanoparticle Inhibit Myeloid-Derived Suppressor Cells. Mol. Cancer.

[116] Lai C., Yu X., Zhuo H., Zhou N., Xie Y., He J., Peng Y., Xie X., Luo G., Zhou S. (2014). Anti-tumor immune response of folate-conjugated chitosan nanoparticles containing the IP-10 gene in mice with hepatocellular carcinoma. J. Biomed. Nanotechnol..

[117] Hu Z., Chen J., Zhou S., Yang N., Duan S., Zhang Z., Su J., He J., Zhang Z., Lu X. (2017). Mouse IP-10 Gene Delivered by Folate-modified Chitosan Nanoparticles and Dendritic/tumor Cells Fusion Vaccine Effectively Inhibit the Growth of Hepatocellular Carcinoma in Mice. Theranostics.

[118] Wang Z., He L., Li W., Xu C., Zhang J., Wang D., Dou K., Zhuang R., Jin B., Zhang W. (2021). GDF15 induces immunosuppression via CD48 on regulatory T cells in hepatocellular carcinoma. J. Immunother. Cancer.

[119] Rosenberg S.A. (2014). IL-2: The first effective immunotherapy for human cancer. J. Immunol..

[120] Tahvildari M., Dana R. (2019). Low-Dose IL-2 Therapy in Transplantation, Autoimmunity, and Inflammatory Diseases. J. Immunol..

[121] Wu J., Tang C., Yin C. (2017). Co-delivery of doxorubicin and interleukin-2 via chitosan based nanoparticles for enhanced antitumor efficacy. Acta Biomater..

[122] Huang K.-W., Hsu F.-F., Qiu J.T., Chern G.-J., Lee Y.-A., Chang C.-C., Huang Y.-T., Sung Y.-C., Chiang C.-C., Huang R.-L. (2020). Highly efficient and tumor-selective nanoparticles for dual-targeted immunogene therapy against cancer. Sci. Adv..

[123] Li J., Lin W., Chen H., Xu Z., Ye Y., Chen M. (2020). Dual-target IL-12-containing nanoparticles enhance T cell functions for cancer immunotherapy. Cell. Immunol..

[124] Tennant D.A., Durán R.V., Gottlieb E. (2010). Targeting metabolic transformation for cancer therapy. Nat. Rev. Cancer.

[125] Warburg O. (1961). On the facultative anaerobiosis of cancer cells and its use in chemotherapy. Munch. Med. Wochenschr. (1950).

[126] Dietl K., Renner K., Dettmer K., Timischl B., Eberhart K., Dorn C., Hellerbrand C., Kastenberger M., Kunz-Schughart L.A., Oefner P.J. (2010). Lactic Acid and Acidification Inhibit TNF Secretion and Glycolysis of Human Monocytes. J. Immunol..

[127] Brand A., Singer K., Koehl G.E., Kolitzus M., Schoenhammer G., Thiel A., Matos C., Bruss C., Klobuch S., Peter K. (2016). LDHA-Associated Lactic Acid Production Blunts Tumor Immunosurveillance by T and NK Cells. Cell Metab..

[128] Raez L.E., Papadopoulos K., Ricart A.D., Chiorean E.G., Dipaola R.S., Stein M.N., Rocha Lima C.M., Schlesselman J.J., Tolba K., Langmuir V.K. (2013). A phase I dose-escalation trial of 2-deoxy-D-glucose alone or combined with docetaxel in patients with advanced solid tumors. Cancer Chemother. Pharmacol..

[129] Sasaki K., Nishina S., Yamauchi A., Fukuda K., Hara Y., Yamamura M., Egashira K., Hino K. (2021). Nanoparticle-Mediated Delivery of 2-Deoxy-D-Glucose Induces Antitumor Immunity and Cytotoxicity in Liver Tumors in Mice. Cell. Mol. Gastroenterol. Hepatol..

[130] Chen Y., Huang Y., Reiberger T., Duyverman A.M., Huang P., Samuel R., Hiddingh L., Roberge S., Koppel C., Lauwers G.Y. (2014). Differential effects of sorafenib on liver versus tumor fibrosis mediated by stromal-derived factor 1 alpha/C-X-C receptor type 4 axis and myeloid differentiation antigen–positive myeloid cell infiltration in mice. Hepatology.

[131] Duda D.G., Kozin S.V., Kirkpatrick N.D., Xu L., Fukumura D., Jain R.K. (2011). CXCL12 (SDF1alpha)-CXCR4/CXCR7 pathway inhibition: An emerging sensitizer for anticancer therapies?. Clin. Cancer Res. Off. J. Am. Assoc. Cancer Res..

[132] Gao D.-Y., Lin T.-T., Sung Y.-C., Liu Y.C., Chiang W.-H., Chang C.-C., Liu J.-Y., Chen Y. (2015). CXCR4-targeted lipid-coated PLGA nanoparticles deliver sorafenib and overcome acquired drug resistance in liver cancer. Biomaterials.

[133] Li G., Liu D., Kimchi E.T., Kaifi J.T., Qi X., Manjunath Y., Liu X., Deering T., Avella D.M., Fox T. (2018). Nanoliposome C6-Ceramide Increases the Anti-tumor Immune Response and Slows Growth of Liver Tumors in Mice. Gastroenterology.

[134] Ngambenjawong C., Gustafson H.H., Pun S.H. (2017). Progress in tumor-associated macrophage (TAM)-targeted therapeutics. Adv. Drug Deliv. Rev..

[135] Wang T., Zhang J., Hou T., Yin X., Zhang N. (2019). Selective targeting of tumor cells and tumor associated macrophages separately by twin-like core–shell nanoparticles for enhanced tumor-localized chemoimmunotherapy. Nanoscale.

[136] Kaps L., Leber N., Klefenz A., Choteschovsky N., Zentel R., Nuhn L., Schuppan D. (2020). In Vivo siRNA Delivery to Immunosuppressive Liver Macrophages by α-Mannosyl-Functionalized Cationic Nanohydrogel Particles. Cells.

[137] Boutilier A.J., Elsawa S.F. (2021). Macrophage Polarization States in the Tumor Microenvironment. Int. J. Mol. Sci..

[138] Dai X., Ruan J., Guo Y., Sun Z., Liu J., Bao X., Zhang H., Li Q., Ye C., Wang X. (2021). Enhanced radiotherapy efficacy and induced anti-tumor immunity in HCC by improving hypoxia microenvironment using oxygen microcapsules. Chem. Eng. J..

[139] Comparetti E.J., Lins P.M.P., Quitiba J., Zucolotto V. (2022). Cancer cell membrane-derived nanoparticles block the expression of immune checkpoint proteins on cancer cells and coordinate modulatory activity on immunosuppressive macrophages. J. Biomed. Mater. Res. Part A.

[140] Li Y., Ayala-Orozco C., Rauta P.R., Krishnan S. (2019). The application of nanotechnology in enhancing immunotherapy for cancer treatment: Current effects and perspective. Nanoscale.

